# Versatile and Easily Designable Polyester-Laser Toner Interfaces for Site-Oriented Adsorption of Antibodies

**DOI:** 10.3390/ijms23073771

**Published:** 2022-03-29

**Authors:** Marcin Drozd, Polina Ivanova, Katarzyna Tokarska, Kamil Żukowski, Aleksandra Kramarska, Adam Nowiński, Ewa Kobylska, Mariusz Pietrzak, Zbigniew Brzózka, Elżbieta Malinowska

**Affiliations:** 1 Chair of Medical Biotechnology, Faculty of Chemistry, Warsaw University of Technology, 00-664 Warsaw, Poland; polina.ivanova@pw.edu.pl (P.I.); mariusz.pietrzak@pw.edu.pl (M.P.); zbigniew.brzozka@pw.edu.pl (Z.B.); 2 Centre for Advanced Materials and Technologies CEZAMAT, Warsaw University of Technology, 02-822 Warsaw, Poland; katarzyna.tokarska@pw.edu.pl (K.T.); kamil.zukowski@pw.edu.pl (K.Ż.); aleksandra.kramarska@pw.edu.pl (A.K.); 3 Section of Inorganic and Analytical Chemistry, Faculty of Chemistry, University of Warsaw, 02-093 Warsaw, Poland; 4 Screenmed Ltd., Okulickiego 5F, 05-500 Piaseczno, Poland; nowinski.adam@gmail.com; 5 Lukasiewicz—Industrial Chemistry Institute, 01-793 Warsaw, Poland; ewa.kobylska.dokt@pw.edu.pl

**Keywords:** protein adsorption, antibody immobilization, hydrophobic interactions, immunosensing, Lab-on-a-Foil, printed microfluidics, C-reactive protein, polyester foil, poly(ethylene terephtalate), printer toner

## Abstract

Laser toners appear as attractive materials for barriers and easily laminated interphases for Lab-on-a-Foil microfluidics, due to the excellent adhesion to paper and various membranes or foils. This work shows for the first time a comprehensive study on the adsorption of antibodies on toner-covered poly(ethylene terephthalate) (PET@toner) substrates, together with assessment of such platforms in rapid prototyping of disposable microdevices and microarrays for immunodiagnostics. In the framework of presented research, the surface properties and antibody binding capacity of PET substrates with varying levels of toner coverage (0–100%) were characterized in detail. It was proven that polystyrene-acrylate copolymer-based toner offers higher antibody adsorption efficiency compared with unmodified polystyrene and PET as well as faster adsorption kinetics. Comparative studies of the influence of pH on the effectiveness of antibodies immobilization as well as measurements of surface ζ-potential of PET, toner, and polystyrene confirmed the dominant role of hydrophobic interactions in adsorption mechanism. The applicability of PET@toner substrates as removable masks for protection of foil against permanent hydrophilization was also shown. It opens up the possibility of precise tuning of wettability and antibody binding capacity. Therefore, PET@toner foils are presented as useful platforms in the construction of immunoarrays or components of microfluidic systems.

## 1. Introduction

The need for cheap and accessible analytical tests for quick self-diagnosis, characterized by a high degree of automation and maximum end-user convenience, is a strong incentive for the development of miniaturized enzymatic immunoassays. The resignation from the classic enzyme-linked immunosorbent assay (ELISA) formats in favor of automated microfluidic systems brings multiple benefits. Material savings (mostly disposable ones) and reduced consumption of biological components comes to the fore, together with normal or only slightly worsened analytical performance [1,2]. The facile implementation of a variety of detection systems based on colorimetric, fluorometric, or electrochemical detection, as well as assays integration into multiplex systems are also obvious advantages [3,4,5].

Hydrophobic interfaces are convenient, passive components for handling liquids, widely exploited in novel microfluidic systems based on microdroplets and spot arrays [6,7]. The advantages of regulated wettability offered by such substrates, such as self-cleaning and barrier properties to a liquid migration, are intensively employed for applications in Lab-on-a-Chip and high-throughput screening formats [8,9]. An interesting issue is also the employment of hydrophobic substrates as interfaces for on-site, passive antibody immobilization. To this day, this approach is considered as a powerful strategy for the formation of immunoreceptor layers in terms of simplicity and reagent consumption [10]. Beyond polystyrene, which is difficult to both process and apply in microfluidic systems (but still remains the gold standard for high-throughput immobilization of biomolecules on microtiter plates), also a number of other materials have been recently reported as immunoassay substrates. These include hydrophobic polymers—more compatible with microfluidic systems and capable of reproduction of complex microarchitectures—such as polydimethylsiloxane (PDMS) [11], thermoplastic polyurethane [12], and fluorinated ethylene-propylene [13], as well as nitrocellulose [14].

Excellent improvement in conventional systems as well as the development of new solutions for PoC diagnostics is offered by the use of technology based on polyester films, which can be easily cut and glued or thermally laminated [15,16]. The two common approaches that are currently trending in Lab-on-a-Foil systems development include: (i) fully “closed” microfluidic systems and (ii) semi-open arrays for high-throughput analytics in microdroplets [17]. In the case of a microfluidic format, the whole system takes the form of disposable cassettes working in the Total Analysis System (TAS) regime [18,19]. In such an integrated cassette, all biochemical reactions as well as various operations related to sample preparation, liquid handling, or signal readout are performed on-site [20,21]. In turn, the semi-open format is characterized by fabrication simplicity and the possibility of performing multiplex measurements, but at the expense of a slightly more complicated assay manual. Flexible and transparent substrates (e.g., foils made of unmodified poly(ethylene terephthalate)) become an excellent alternative to solid polymer substrates (thermoplastics, PDMS) or glass, both at the stage of rapid prototyping and mass production [22]. Easy scalability of the laser-cutting and gluing/lamination technology allows the production of flexible microfluidics both in short series for the needs of R&D and industrial formats, e.g., in the roll-to-roll technology [23].

All the tools and methods for rapid prototyping of cheap and disposable fluidic systems with easily available methods are currently trending under the name “Do It Yourself” (DIY) microfluidics [24]. One of its main branches is based on the use of flexible substrates, such as polyester foils and direct contact printing. So far, laser toner has found applications, i.a., as a hydrophobic barrier for valves and droplet handling [25,26] and a material for formation of microstructures, as well as a thermo-responsive adhesive that enables lamination [27,28]. A number of microfluidic analytical systems using PET-toner interfaces have been developed for applications such as capillary electrophoresis [29], separation of nucleic acids [30,31], and chemical and biochemical assays (using colorimetric or enzymatic reactions) for the determination of relevant analytes [32,33,34,35]. Until now, laser toner has not been considered in a construction of immunoassays as a readily applied, hydrophobic substrate for the direct, passive immobilization of antibodies. The presented strategy is very promising and meets global trends and growing demand for fast and easily designable immunodiagnostic tools. Recently, toner as well as hydrophobic waxes and curable varnishes have been employed as a support in the construction of lateral flow immunoassays and ELISA arrays on PET foils [9,25,27,32,34]. Laser toner is emerging as a very cheap and convenient intermediate for antibodies adsorption. Laser printing may surpass dispensing of carbon nanomaterials (i.a., graphene derivatives or nanotubes) in Lab-on-a-Foil assays fabrication in terms of availability of tools for rapid prototyping and large-scale production [36,37,38]. In this study, we report for the first time a novel approach in which flexible PET substrates covered with a laser toner were used as platforms for passive antibody coating. For this aim, we took advantage of high antibody binding capacity of originally hydrophobic toner and proved its applicability as a removable mask, locally protecting the surface against dry hydrophilization. We evaluated the influence of toner coverage (expressed as printing at various grayscale levels) on both the total amount of immobilized antibodies and their affinity towards human C-reactive protein (CRP) as model antigen. Moreover, other physicochemical and surface parameters of as-obtained PET@toner interfaces, such as surface wettability and charge, were characterized and benchmarked against pristine PET and polystyrene. We demonstrated the possibility of fine-tuning of PET@toner properties using easily accessible methods based on selective laser printing and printed toner removal. It was confirmed that PET@toner substrates show a number of interesting functionalities from the point of view of spatially resolved antibody immobilization and construction of platforms for high throughput screening or liquid handling for microfluidics.

## 2. Results and Discussion

**Scheme 1:**
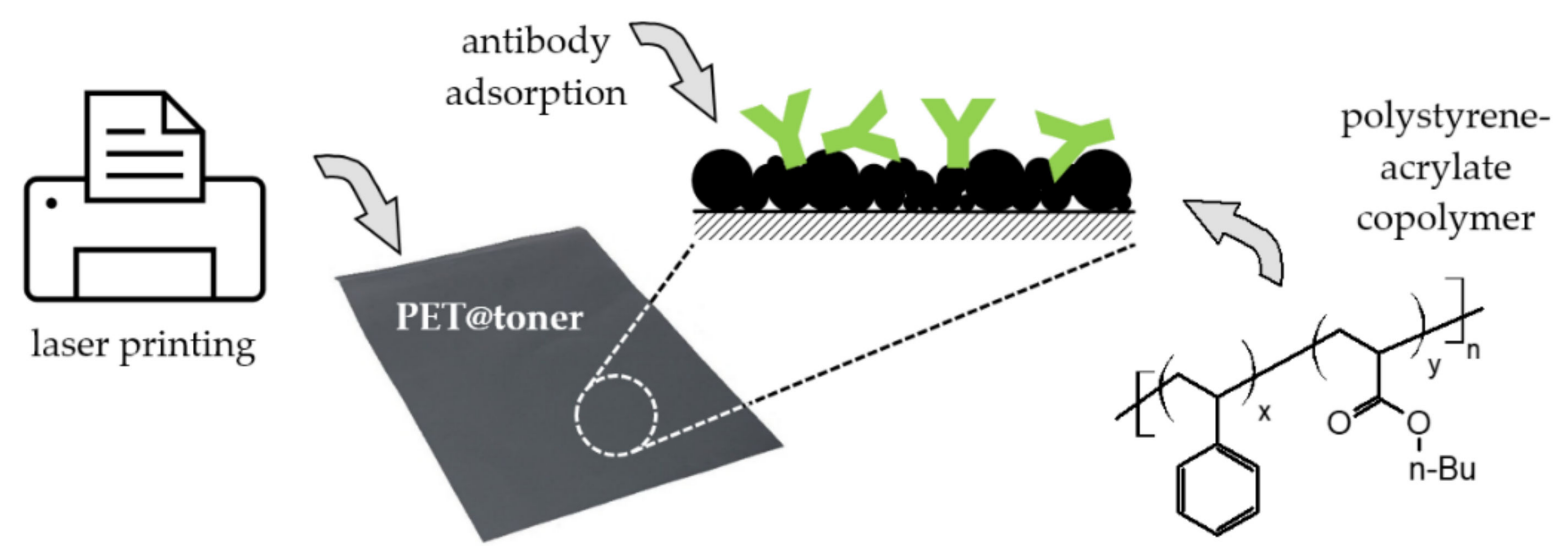
Schematic illustration of the fabrication process of PET@toner-based substrates for antibodies immobilization and immunosensing. PET—poly(ethylene terephtalate), n-Bu—*n*-butyl.

Due to the ease of coating with toner by direct printing and the capability of rapid laser processing, as well as the continuous, pore-free, and waterproof structure, unmodified PET foil for laser printers was chosen as the model substrate for the laser toner-based platforms. Scheme 1 graphically shows the concept of application of a cured toner as interphase for the fabrication of immunoassay on a flexible substrate. The process covers contact-printing of an appropriately designed toner pattern on the polyester foil intended for laser printers, which determines the final properties of PET@toner surface. In our case, for the characterization of printed surfaces with different degrees of toner coverage, a uniform, black overprint at various grayscale levels (0–100%) was used. Laser printing resulted in obtained a thin (thickness of the toner layer amounted to about 2.6 µm) and flexible layer characterized by excellent adhesion to the polymeric foil. Due to the dominant content of polystyrene-based copolymer in the toner composition, it can be expected that the obtained interfaces will show properties similar to polystyrene—in particular, very good capacity for antibody binding driven by passive adsorption. The mechanism of applying toner onto PET foil uses thermal curing of polystyrene-acrylate copolymer powder, which guarantees plasticization of microspheres and their adhesion to the polyester film [39]. As a result, stable coatings of fused toner are formed on the PET surface, additionally stabilized by hydrophobic interactions. In turn, the strong adsorption of antibodies on the hydrophobic toner surface is caused by the interaction of Van der Waals forces and the interaction of hydrophobic domains of glycoprotein chains, especially *β*-sheets rich in aromatic amino acids. The mechanism is probably similar, as is the case with other polymeric substrates, such as PS, PTFE or silicone [40].

### 2.1. Morphological and Optical Properties of the PET@Toner Surfaces

**Figure 1:**
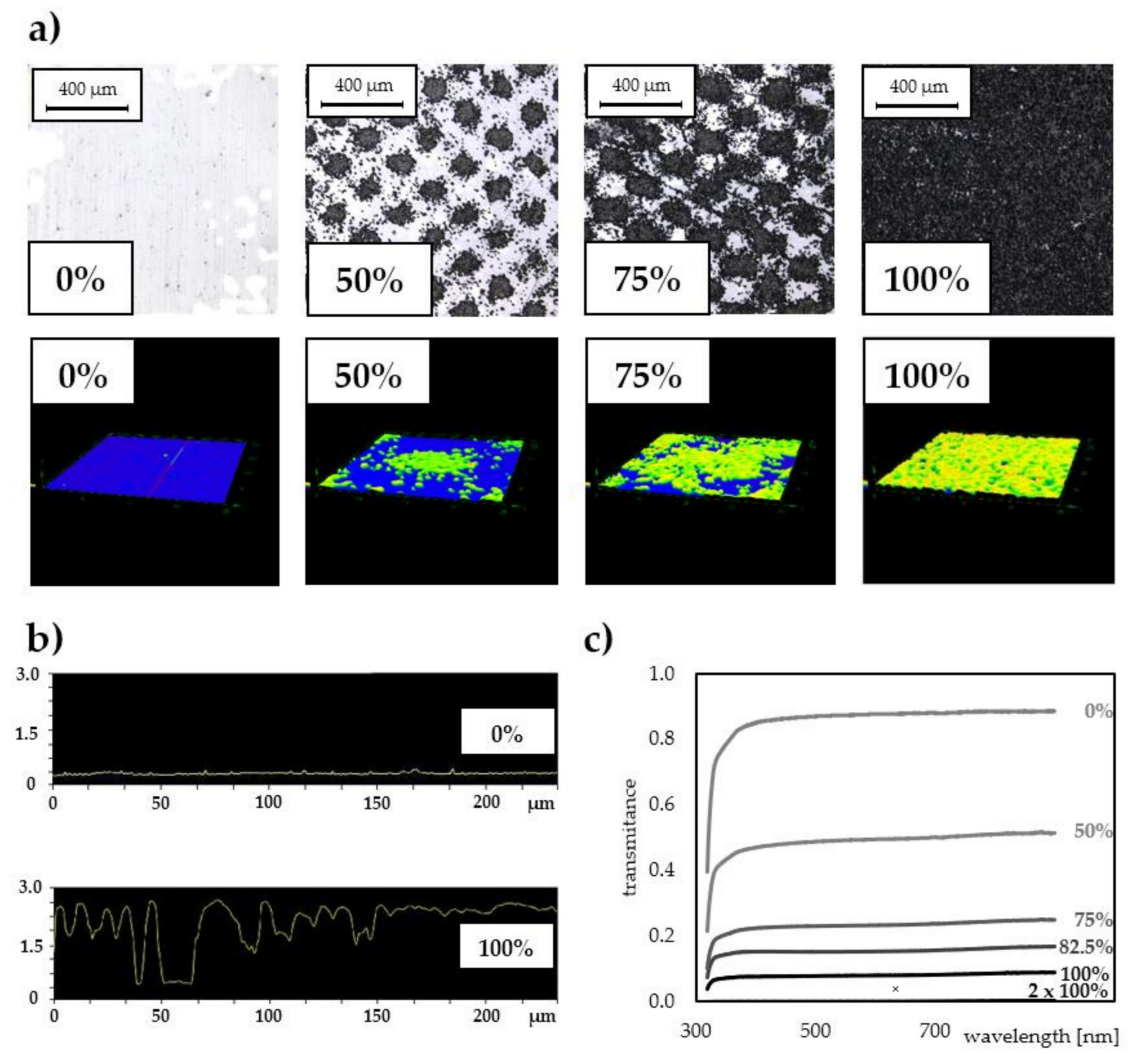
(**a**) Optical micrographs of toner surfaces printed on PET foils at different grayscale levels (0–100%) (**top row**); topographies of single toner islets at higher magnification imaged in 3D with a laser microscope (**bottom row**). (**b**) Cross-sectional profiles of PET (pristine) and PET@toner at 100% grayscale level. (**c**) Transmittance spectra of toner-covered foils printed at various grayscale levels (0%, 50%, 75%, 82.5%, 100%, 2 × 100%) registered in the range 300–900 nm.

Laser printing with the use of a mid-class office laser printer working in a resolution of 600 dpi enables easy reproduction of predesigned pattern with relatively high fidelity, in which details are well below 100 μm. In addition, the function of printing in a grayscale allows the fabrication of regularly spaced, hydrophobic toner microplates. Micrographs obtained with a laser microscope (Figure 1a) display architectures of PET substrates covered with the toner using different grayscale levels. Visible islands of grained toner thermally cured with polystyrene-acrylate copolymer gradually cover the increasing foil area, progressively cutting off the access to PET surface (Figure 1a, top row). In turn, obtained 3D images (Figure 1a, bottom row) and profiles (Figure 1b) illustrate the shape and height of toner microstructures on the foil surface, respectively. It was confirmed that the laser toner layer on the foil is water-resistant. Moreover, it exhibited resistance to exposure to polar organic solvents (isopropanol, acetone). Additionally, thanks to the small thickness, PET@toner interfaces form both durable and tightly adherent arrangements with typical adhesive tapes, which also makes them compatible with the technology of flexible microfluidics (Lab-on-a-Foil). This property was exploited in the later stages of presented studies for the fabrication of test microwells on PET@toner substrates in the technology of laser cutting and gluing.

Due to the partial coverage of the carrier surface with a toner, it is possible to fine-tune its optical properties such as transparency in the Vis–NIR range. The scattering of the beam passing through the partially toner-covered foil does not lead to a complete loss of transparency, even at relatively high grayscale levels, as shown in Figure 1c. For example, even at a blackness level of 82.5%, the transmittance of PET@toner substrate remains above 10% (which corresponds to background absorbance < 1), and for lower grayscale levels the contribution of background scattering is correspondingly less significant.

### 2.2. The Influence of Toner-Based Substrates on Efficiency and Kinetics of Antibody Binding

**Figure 2:**
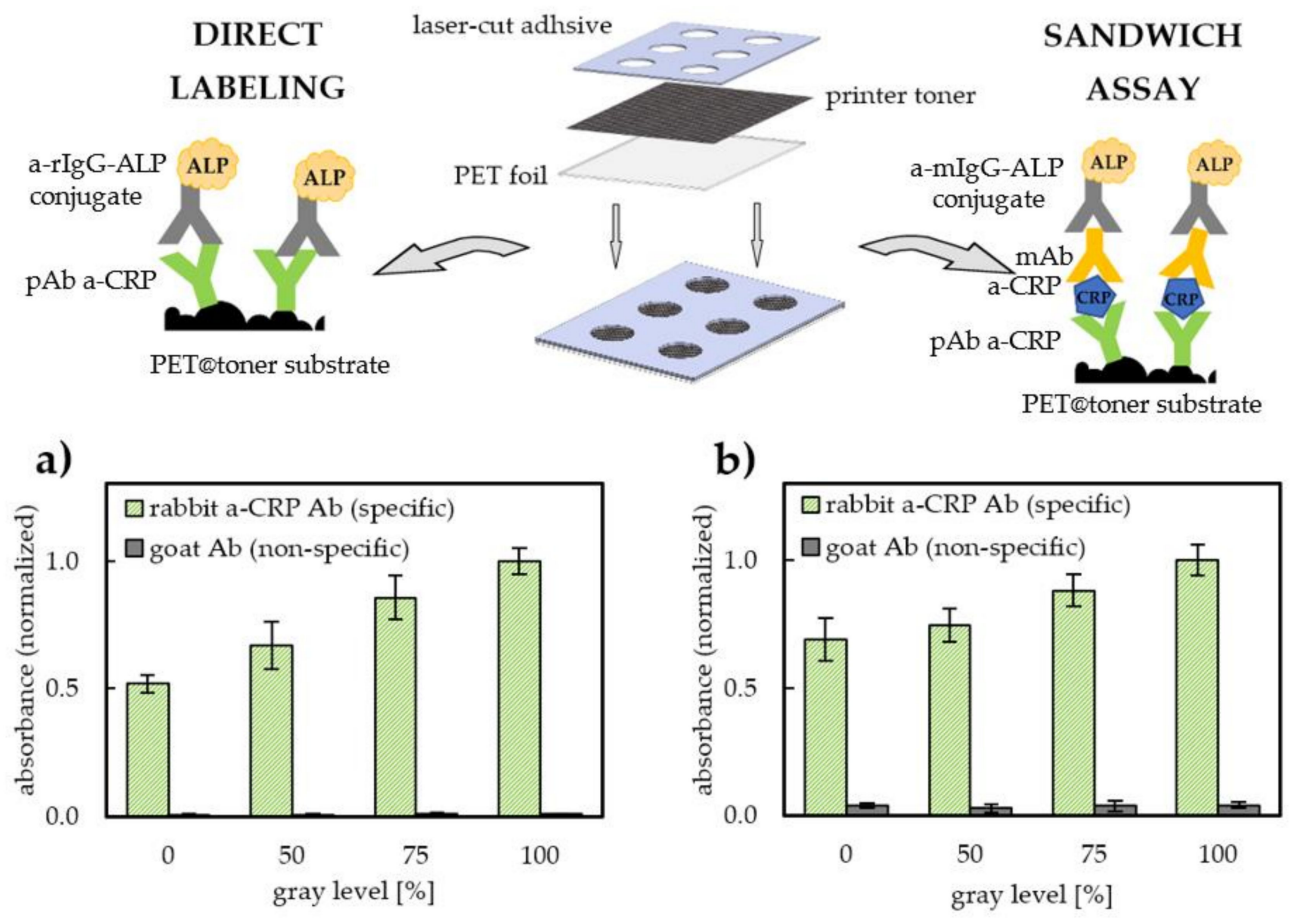
Top row: representation of the fabrication process based on the laser-printing of PET foil and microwell formation using specially designed laser-cut adhesive tape (**center**). Schematics of ELISA formats used within this study for the comparative evaluation of: (i) antibody surface density (**left**—antibody immunolabeling by means of direct ELISA), (ii) affinity of immobilized Abs towards C-reactive protein used as model antigen (**right**—indirect sandwich ELISA). Bottom row: relative surface density of adsorbed pAb a-CRP (2 h immobilization, 5 µg/mL Abs solution in carbonate buffer pH 9.6) on PET@toner substrates printed at different grayscale levels (*n* = 5). Values are expressed as absorbance signals in the result of Abs immunolabeling with a-rIgG-ALP as conjugate and *p*-nitrophenyl phosphate (PNNP) as a substrate (**a**), relative sensitivity of toner-based sandwich immunoassays (CRP concentration = 100 ng/mL, 30 min incubation) on PET@toner substrates printed at different grayscale levels (*n* = 5) (**b**). a-rIgG—anti-rabbit IgG antibody, ALP—alkaline phosphatase, pAb a-CRP—polyclonal antibody against C-reactive protein, PET—poly(ethylene terephtalate), a-mIgG—anti-mouse IgG antibody, mAb a-CRP—monoclonal antibody against C-reactive protein.

To evaluate the applicability of laser toner-coated PET as a substrate for immobilization of antibodies, first the effect of the degree of toner coverage on both the antibody binding capacity and ability to bind model antigen (C-reactive protein, CRP) was checked. Utilization of hydrophobic interactions remains very popular and is widely used as a driving force of passive immobilization of immunoglobulins, which is exemplified by the routinely used antibody-coated microtiter plates, acting as supports in a construction of diagnostic immunoassays. Hence, it was decided to compare the ability of antibodies to be immobilized on an unmodified (originally hydrophobic) PET substrate and printed micropatterns obtained using the laser toner. For this purpose, foils printed at various grayscale levels (0–100%) were used. To ensure the uniformity of the tested geometric areas, a laser-cut masks made of a medical adhesive tape were attached to the previously printed PET@toner pieces. In such a way, two-layer, semi-open arrays of microwells with an architecture corresponding to a multi-well plate were obtained (See Figure 2). This format seems to be perfectly suited to immunoreactions carried out in high-throughput in droplet-arrays. The application of enzymatically labeled antibodies (goat-anti-rabbit IgG conjugated with alkaline phosphatase, a-rIgG-ALP), followed by detection of a water-soluble chromogenic reaction product, allowed for a comparison of the relative surface loadings of rabbit polyclonal anti-CRP antibodies (pAb a-CRP) used as a model immunoreceptor. On the other hand, an indirect sandwich assay was constructed to evaluate the total antigen-binding capacity of adsorbed pAb a-CRP. Details of the construction of immunoassays used in this study are schematically presented in Figure 2.

The obtained results of immunolabeling and conducted sandwich assay confirm that it is possible to effectively immobilize antibodies directly on the surface of the laser toner. Based on the results depicted in Figure 2a,b, we can observe that both the total amount of capture antibody (pAb a-CRP) and total amount of antigen bound per single spot (representing assay sensitivity) increase with the increase in the PET film coverage with the toner. Relative surface density of immobilized antibodies increases in a manner close to linear, reaching a value nearly 2 times higher for full coverage (100% grayscale level) compared with pristine PET foil (increase in normalized Ab coverage from 51.8% maximum to 100%, see Figure 2a). We can assume that the sources of the observed phenomenon derive from both the increased specific surface area of a toner surface (due to the natural roughness of the cured toner layer) and high antibody binding capacity of the laser toner. It is worth emphasizing that rough toner surface does not contribute to the increase in non-specific protein adsorption, which is manifested by the negligibly low level of non-specific binding of conjugate on control fields coated with immunoglobulin G from goat (gIgG) (used as a negative control). The contribution of non-specific adsorption did not exceed 1.2% for direct immunolabelling and 4.0% for sandwich assay (see Figure 2a,b). Immobilization of antibodies on PET and PET@toner was also confirmed by local immunostaining using precipitating ALP substrate BCIP/NBT. The specific staining of the zones previously coated with antibodies confirms the effective and stable adsorption during the immunoassay procedure.

Despite the increased surface area and more heterogeneous and grained topography of PET@toner, the surface blocking and washing-off of unbound conjugate turned out to be as effective as for pristine PET. The relation of the sensitivity of CRP-sandwich immunoassay in the function of the degree of toner coverage (Figure 2b) shows a similar character to the relation representing the total amount of adsorbed antibody. The signal intensity of indirect sandwich assay on a pristine PET corresponds to 69% of the intensity of a fully printed substrate. This observation confirms that antibody immobilization on the toner does not significantly deteriorate the affinity of immobilized antibody to its antigen in comparison with PET. The total antigen-binding capacity of antibodies immobilized on the toner surface is slightly lower than for PET film, as evidenced by the lower ratio of sandwich assay sensitivities for 100% and 0% grayscale levels (100%/69% = 1.45), in comparison with a similar ratio calculated for the total amount of antibodies (100%/52% = 1.92). This can be explained by the increased susceptibility to conformational changes of antibodies due to interactions with the strongly hydrophobic toner. Binding to a strongly hydrophobic surface entails the risk of protein unfolding and exposition of hydrophobic domains, resulting in a partial denaturation, which is a typical phenomenon observed for passive adsorption of antibodies [41,42]. The reported degree of Abs denaturation may reach about 50%, which derives from protein conformational changes and random immobilization, hindering solvent accessibility of binding sites [43]. It is however worth underlining that in our case this effect is fully compensated for by the benefits resulting from the increased surface area. For the examined pAb a-CRP immobilization conditions, no negative effects related to the appearance of steric hindrances were found. Deteriorated antigen binding in the case of high antibody surface coverages can be observed in the case of other hydrophobic immunoassay substrates [13]. The main advantage of laser toner compared with hydrophobic carbon nanomaterials, e.g., graphene or carbon nanotubes, lies primarily in their great availability and low toner price. It is worth emphasizing that the facility of application on the PET substrate is because the organic–inorganic copolymer composite with iron oxide powder is in the form of a ready-to-use and commercially available formulation and that the printing process by means of thermal curing is possible with the basic office device. In turn, in the case of carbon nanomaterial-based pastes and inks applied on PET substrates, it is necessary to use dedicated screen printing or spray coating tools [44,45].

**Figure 3:**
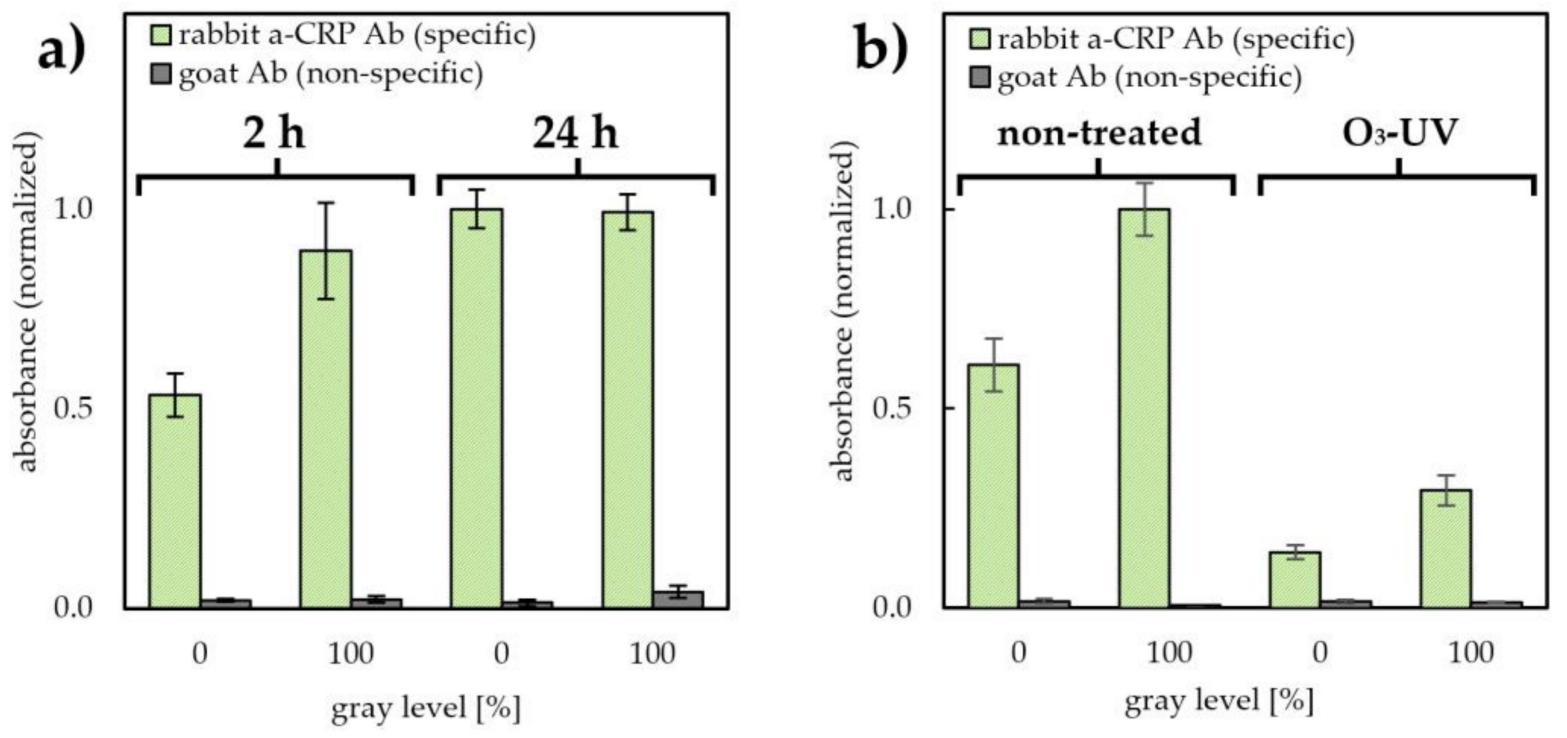
Comparison of the kinetics of antibody adsorption on PET and PET@toner (100% grayscale level) after 2 h and 24 h antibody coating (5 µg/mL pAb a-CRP Abs solution in carbonate buffer pH 9.6, *n* = 5) (**a**). Studies on the effect of dry hydrophilization (5 min ozone/UV treatment) on pAb a-CRP antibody binding capacity of PET and PET@toner substrates (*n* = 5) (**b**). pAb a-CRP surface densities were determined by direct immunolabeling by means of 30 min incubation in 500 ng/mL a-rIgG-ALP conjugate in PBST buffer with 1% bovine serum albumin (BSA). Values are expressed as relative absorbances of the reaction product derived from 15 min immunolabeling. Ab—antibody, a-CRP Ab—antibody against C-reactive protein.

In the next step, the influence of the substrate type on the kinetics of antibody adsorption was investigated. For this purpose, the antibody surface density on PET and PET@toner (100% grayscale level) were compared. Examined conditions of immobilization covered the most typical procedures used for antibody coating described in the literature when using microtiter plates—i.e., 2 h in ambient temperature and 24 h (overnight) in 4 °C. It was observed that the type of the substrate used influences the relative rate of antibody binding (Figure 3a). Interestingly, after 2 h of surface coating (immobilization time typically applied within presented studies) more efficient immobilization has been noticed on PET@toner. This observation stays in line with the results discussed in Figure 2 and the results obtained by Barbosa et al., where a surprisingly fast rate of reaching equilibrium during the adsorption of antibodies was also observed on the strongly hydrophobic fluoropolymer surface [13]. It is noteworthy, that elongation of the immobilization time to 24 h resulted in the suppression of the observed differences. After overnight immobilization, density of antibodies on the toner and PET were practically the same. This fact indicates that the toner proved to be a valuable substrate for passive protein immobilization, especially in the case of technologies that impose the requirement of a relatively short immobilization time.

### 2.3. Studies on the UV-Induced Ozone Oxidation as a Dry Approach to Toner Hydrophilization

Rapid fabrication of a permanent, locally hydrophilic pattern on flexible substrates is particularly desirable, both due to the possibility of obtaining anti-fouling surfaces resistant to interactions with proteins, as well as high wettability of such zones. This opens up the possibility of designing substrates with spatially adjusted hydrophilicity for novel microfluidic systems based on flexible components. Therefore, we have validated the effectiveness of the ozone/UV treatment as a dry approach of PET and PET@toner hydrophilization. This was accomplished by a brief (5 min) ozone-mediated oxidation induced by UV irradiation. As can be seen in Figure 3b, after hydrophilization, a significant decrease in the amount of immobilized antibodies is observed on both types of substrates. The relative decrease in antibody surface density was slightly greater for pristine PET (ratio between non-treated (n-t) and treated foil amounted to 4.35, in comparison with 3.38 for PET@toner at 100% grayscale level). These observations are supported by literature reports according to which, the increased surface wettability counteracts protein adhesion [46].

Moreover, we thoroughly investigated and demonstrated that it is possible to further improve the anti-fouling properties by extending the exposure to ozone/UV irradiation. For example, extending the oxidation time to 30 min allowed a ratio of about 8.3 to be achieved, but it took place at the expense of deteriorating mechanical properties of the toner layer. Increased fragility of cured toner was observed, which was presumably caused by substantial damage to the copolymer structure arising from its deep oxidation. High resistance of cured toner towards non-specific adsorption of immunoglobulins (both after “wet” protocol of surface blocking as well as “dry” hydrophilization) seems particularly important in light of recent reports in which low-affinity serum components (e.g., autoantibodies) are indicated as the main cause of the serum matrix effect [47].

**Figure 4:**
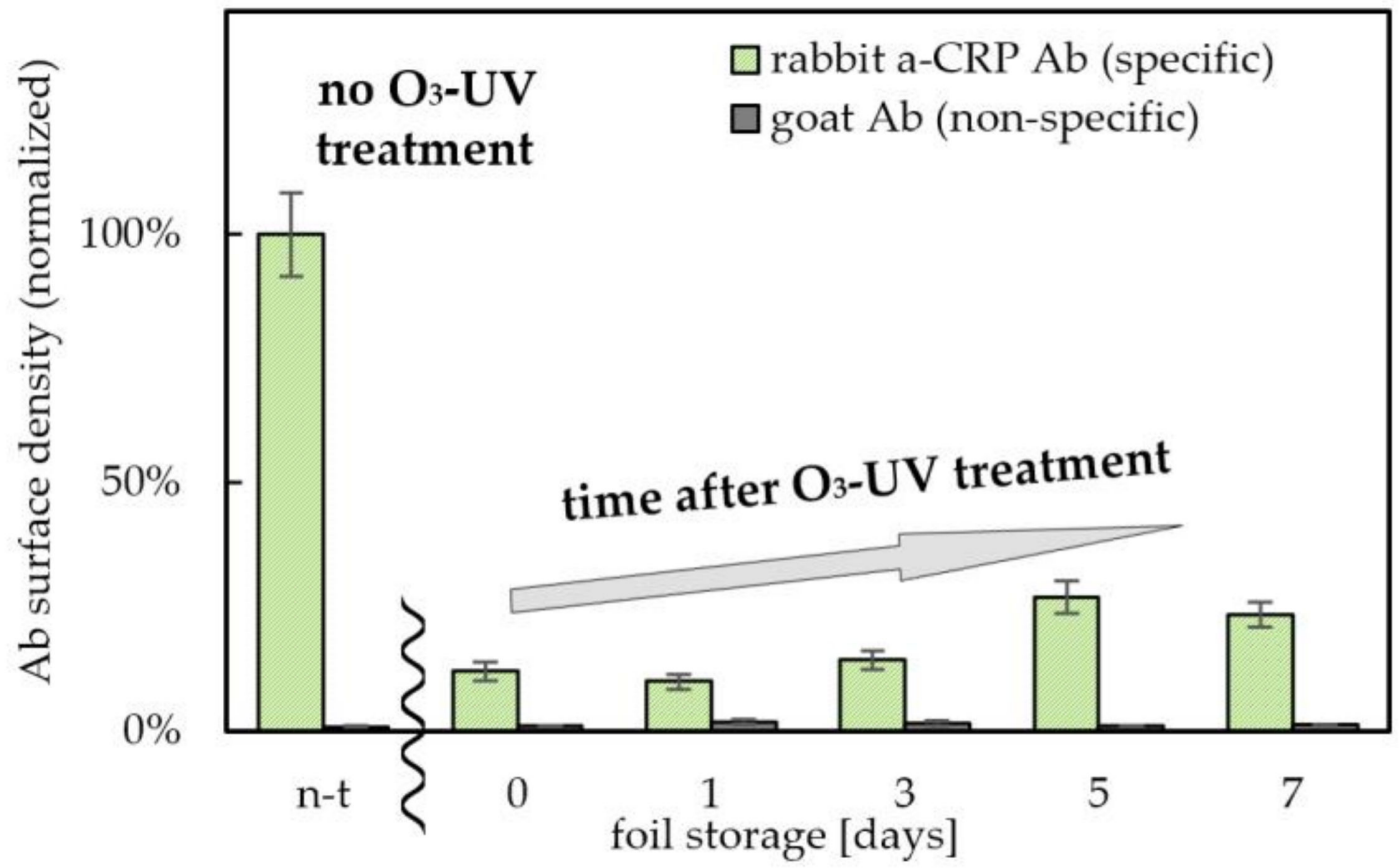
Long-term stability of the effect of ozone/UV treatment (30 min irradiation) on PET@toner (100% grayscale level). The number of days represents the time between surface hydrophilization and antibody immobilization and immunolabeling. Relative densities of pAb a-CRP were calculated based on absorbances of enzymatic reaction product obtained by labeling of adsorbed antibodies with 500 ng/mL a-rIgG-ALP conjugate in PBST buffer with 1% BSA and further reaction with 10 mM PNNP substrate in 50 mM carbonate buffer, pH 9.6. Ab—antibody, a-CRP Ab—antibody against C-reactive protein, n-t—non-treated, a-rIgG—anti-rabbit IgG antibody, ALP—alkaline phosphatase.

The key parameter from the point of view of the proposed technology is the long-term stability of the obtained layers. Maintenance of high wettability and low binding capacity of hydrophilized zones under storage conditions is particularly important in the context of the implementation of PET@toner substrates in microfluidic devices. Such zones can find applications in the construction of layers responsible for transportation of reagents, e.g., in form of microchannels. Therefore, in the current experiment, long-term stability of anti-fouling properties of toner-covered PET foils after ozone/UV irradiation was tested, and the results were normalized to the non-hydrophilized PET@toner substrate. As shown in Figure 4, the amount of adsorbed pAb a-CRP to the hydrophilized PET@toner foils remained relatively low for at least 7 days after irradiation. The highest anti-fouling capacity is shown by the foil irradiated immediately before the Ab coating (12.0% of the initial value). Oxidized PET@toner foil stored in a dry environment for 5–7 days after hydrophilization partially lost its anti-fouling properties (Ab surface density corresponding to 23–27% of the value for the unmodified PET@toner substrate was measured). It is however worth underlining that even then there was still about a 4 times lower amount of adsorbed antibodies than for the substrate before the hydrophilization. These results prove the permanent oxidation of the toner surface with the formation of stable, hydrophilic groups counteracting the adsorption of protein components. This distinguishes the toner from another popular material used in microfluidic technologies, i.e., PDMS, for which the hydrophilic properties of the surface decay spontaneously in a short time [48].

### 2.4. Fine Adjustment of Wettability and Antibody Binding Capacity of PET Foil by Toner Masking

**Figure 5:**
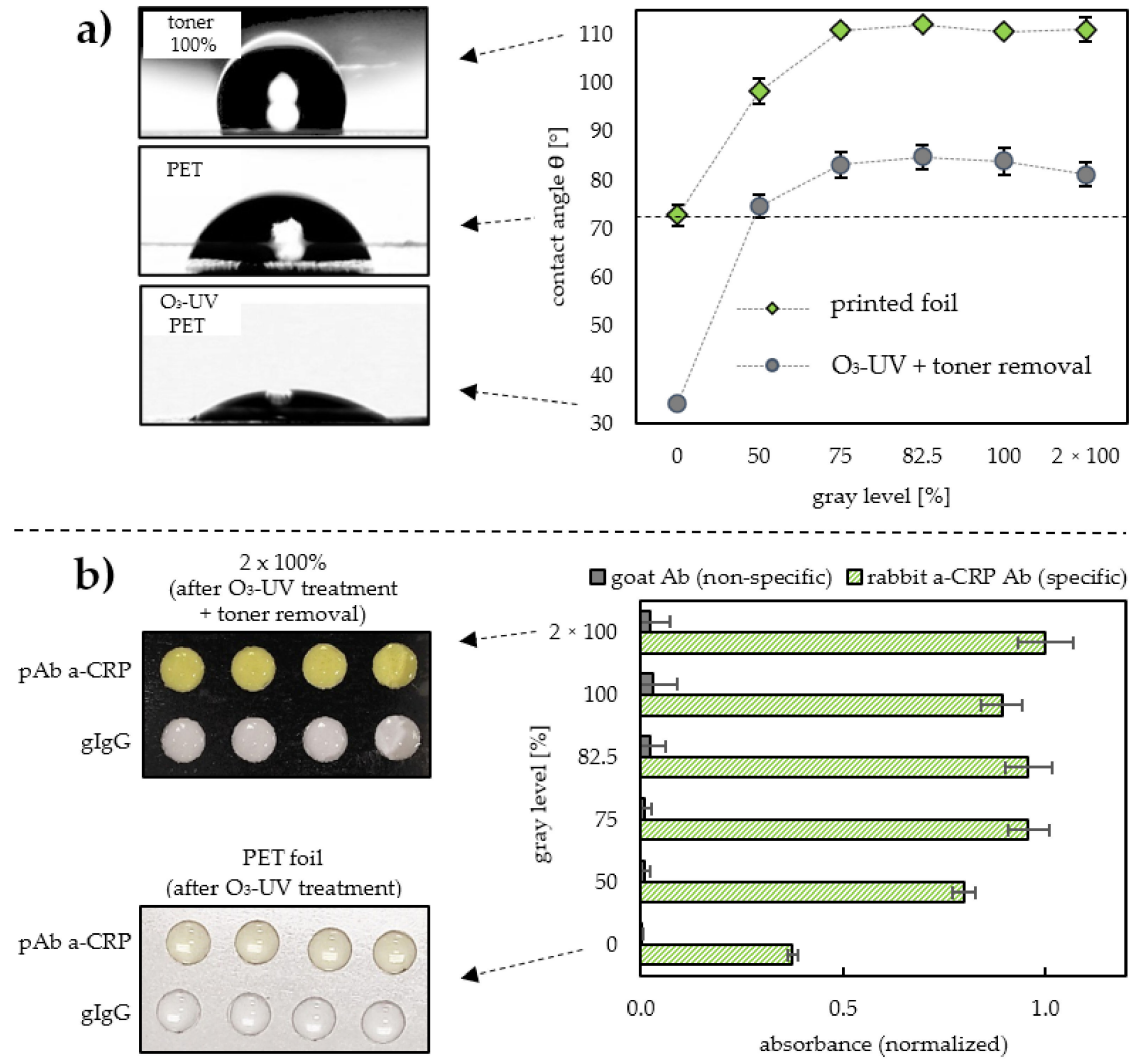
Evaluation of the masking capacity of cured laser toner in terms of preventing dry hydrophilization (by means of ozone/UV treatment) of the PET backing and gaining anti-fouling capability against pAb a-CRP adsorption. (**a**) Water contact angle properties of PET@toner substrates (printed at various grayscales) and their derivatives after irradiation and toner removal (*n* = 6). Non-irradiated PET@toner substrates (pale green dots in Figure 5a) were compared with analogous PET@toner substrates subjected to 10 min ozone/UV treatment followed by toner removal (gray dots). (**b**) Exemplary images and results of specific immunolabeling of adsorbed pAb a-CRP (2 h immobilization, 5 µg/mL Abs solution in carbonate buffer pH 9.6, gIgG used as reference) on various PET@toner surfaces after ozone/UV treatment and toner removal (*n* = 5). Experiment was carried out by labeling of adsorbed antibodies with 500 ng/mL a-rIgG-ALP conjugate in PBST buffer with 1% BSA and further reaction with 10 mM PNNP substrate in 50 mM carbonate buffer, pH 9.6. PET—poly(ethylene terephtalate), gIgG—goat IgG antibody, pAb a-CRP—polyclonal antibody against C-reactive protein.

Excellent toner adhesion to the foil, as well as the possibility of its facile removal through a brief sonication in organic solvent, paves a way to use this material as a mask locally protecting PET backing underneath against hydrophilization. In addition, the above-described print at various grayscale levels seems to be especially attractive from the point of view of the fine-tuning of surface properties, as it opens up the possibility of adjustment of both the wettability and antibody binding capacity. It seems to be achievable by printing of PET, which—depending on the applied grayscale level—can selectively protect part of the backing foil against oxidation during ozone/UV treatment. To verify this hypothesis, investigations on the effectiveness of toner application in a role of a removable mask were carried out. To evaluate masking properties of the toner, a series of contact angle measurements were carried out, the results of which are shown in Figure 5a. Wettabilities of two sets of PET@toner substrates with various degrees of toner coverage were compared. The first set had not been subjected to ozone/UV treatment, and the second set had been at first subjected to 5 min irradiation followed by toner removal. Contact angle values for non-irradiated PET@toner substrates increased with the tested grayscale levels, ranging from 73° (0% coverage) to 111° (75% coverage), and a further increase in the grayscale level did not lead to any increase in the substrate hydrophobicity. As expected, laser toner turned out to be highly hydrophobic, with a wettability comparable with materials such as PDMS (~110°) [49,50] and poly(tetrafluoroethylene) (PTFE) (107°) [51]. Toner hydrophobicity turned out to be far superior to pristine PET foil, which is easily noticeable on sessile drop photographs (See Figure 5a).

An interesting relationship can be observed in the case of PET backings after UV irradiation followed by a toner removal. Already starting from 50% grayscale level, a significant increase in the contact angle was observed, compared with the unprinted foil subjected to ozone/UV treatment (increase from 34° to 74°). It is worth noting that for the coverage amounting to 50% (and higher), PET foil wettability after subsequent masking with the toner and UV irradiation was lower than for pristine PET. The surprisingly high efficiency of the toner as a removable mask, even in the case of incomplete PET backing coverage, can be explained by its remarkable hydrophobicity. As can be seen, for substrates after UV irradiation and toner removal, and with a coverage level > 50%, the values of contact angles exceeded 80°, which was much higher than the angle measured for pristine PET (also subjected to an analogous process simulating toner removal). This increase in hydrophobicity may be due to the occurrence of micro-residues of toner waxes (invisible to the naked eye after toner removal), or—more likely—to the phenomenon of foil peeling/scratching with toner grains during laser printing and its ultrasonically assisted removal. As a result, freshly exposed areas of the PET surface give the entire substrate a more hydrophobic character. The obtained results clearly demonstrate the effectiveness of the masking properties of the laser toner.

In order to comprehensively evaluate the effectiveness of the toner as a removable mask in fine-tuning of PET surface properties, the results of the wettability measurements were confronted with the determination of antibody binding capacity. PET@toner substrates printed at various grayscale levels were subjected to 5 min irradiation followed by toner removal and then used for passive immobilization of antibodies. The results of immunolabeling presented in Figure 5b are undoubtedly correlated with the conclusions drawn from the contact angle measurements, as a strong effect of toner masking could also be observed for the grayscale levels from 50%. It was manifested by a more than 2-fold increase in the amount of immobilized antibodies for the PET@toner substrate primary printed at 50% grayscale, in comparison with the pristine PET. Moreover, as in the case of wettability, a plateau of the masking effect was also observed around 75% of coverage, which guarantees a constant, close to the maximally achievable surface density of the antibodies. For higher grayscale levels, a slightly increased level of non-specific interactions (grey bars in Figure 5b) was observed. However, the total contribution of such effect in the obtained analytical signal was still negligible and did not exceed 3.2%, which proved that the surface was quite effectively protected against protein fouling.

### 2.5. Assessment of the pH of the Medium and the Substrate Type on the Mechanism and Efficiency of Antibody Coating

The observed differences in kinetics and protein binding capacity became the reason for a more detailed, comparative characterization of passive adsorption mechanism on various surfaces. To assess the contribution of electrostatic interactions, surface charges (surface ζ-potentials, SZP) were determined for three types of surfaces (PET foil, toner-covered PET foil, and polystyrene). For measurements, two typical media compositions used in ELISA protocols for passive antibody coating—i.e., 50 mM carbonate buffer pH 9.6 and 50 mM acetate buffer pH 5.0—were employed [52]. Such a choice was dictated by a need to ensure the differentiation of antibodies charge, as the pI of pAb a-CRP is approximately in the range typical for IgGs, between 6.1 and 8.8 [53]. In order to better understand the mechanism of antibody binding, the SZPs were determined for both the unmodified substrates and substrates previously coated with antibodies. Detailed research results are summarized in Table 1.

Studies conducted in parallel on the antibody adsorption showed a little pH-dependency of the coating efficiency for poly(ethylene terephtalate) (PET) and polystyrene (PS) substrates, and only a slightly higher one for toner (a decrease of about 14.5 percent with a decrease in pH from 9.6 to 5.0). PET and PET@toner substrates exhibited a relatively high total antibody binding capacity—nearly 50% higher than the Ab surface density obtained for the unmodified polystyrene substrate used as a reference. Typical surface density of antibodies adsorbed on untreated PS is 100–200 ng/cm^2^, so it can be easily assumed that for PET and PET@toner substrates surface density reaching ~150–300 ng/cm^2^ is achievable. This makes both types of flexible substrates examined in the framework of this study similar to other hydrophobic surfaces described in the literature, such as Teflon amorphous fluoropolymer (Ab binding capacity ~200 ng/cm^2^) [54] or decanethiol-based self-assembled monolayer on gold (468 ng/cm^2^) [42], but with a remarkably simpler fabrication and unit cost.

The strongly anionic character of all studied materials—pristine PET, toner, and polystyrene, both before as well as after the adsorption of antibodies—suggests that in all cases the impact of electrostatic forces is negligible and that the main driving force of antibodies assembly are hydrophobic interactions. The adsorption-powered increase in the entropy due to the release of protein-associated water molecules and ions compensates for the forced conformational changes and results in a strong interaction with hydrophobic surfaces [13]. The undiminished adsorption of negatively charged antibodies on highly anionic substrates at pH 9.6 (SZPs between −40 and −50 mV) indicates a negligible disturbing effect of electrostatic repulsion. What is more, the emergence of antibody adsorbates in the medium both below (pH 5.0) and significantly above the isoelectric point (pH 9.6) does not significantly alter the surface charge. Surprisingly, in each case, pAb a-CRP contributed to a further decrease in the surface ζ-potential by several mV, depending on the substrate used.

The results presented in Table 1 for all types of examined surfaces confirm the similarity of the mechanisms governing the process of adsorption of model immunoglobulins. Thus, the PET and PET@toner substrates can be assigned to the broad family of substrates for passive immobilization of antibodies, which are characterized by significant hydrophobicity and a strongly anionic character in antibody coating medium. Flagship examples of such materials are polystyrene and PDMS or PTFE. They were already employed in the construction of modern immunoassays and biosensors, both in a form of flat surfaces and microstructured dispersion in a role of immunobeads. These examples justify the applicability of passive adsorption as a robust, simple, and efficient approach to fabrication of immunosensing platforms.

## 3. Materials and Methods

### 3.1. Reagents and Solutions

Polyclonal antibodies rabbit anti-human C-reactive protein, 5 mg/mL (pAb a-CRP); goat IgG antibodies, 1 mg/mL (gIgG); polyclonal goat antibodies anti-rabbit IgG conjugated with alkaline phosphatase (a-rIgG-ALP), 1 mg/mL; polyclonal goat antibodies anti-mouse IgG conjugated with alkaline phosphatase (a-mIgG-ALP), 1 mg/mL; and bovine serum albumin were purchased from Sigma-Merck. Mouse monoclonal antibodies anti-human C-reactive protein, 2 mg/mL, clone 6404 (mAb a-CRP), were from Medix Biochemica. C-reactive protein, 3.1 mg/mL, was from ProSpec. All non-biological reagents used in this study were at least of analytical grade.

### 3.2. Preparation of PET and PET@toner Substrates and Antibody Coating

#### 3.2.1. Fabrication of Various PET-Based Substrates and Microwell Arrays for Immunoreactions

Unmodified PET sheets in A4 format, used as substrates for antibody adsorption or laser printing, were purchased from Argo (Poland). Polystyrene (PS) substrates were from ChemLand (Stargard, Poland). HP LaserJet 1006 laser printer working at 600 dpi print resolution was employed for toner printing at various grayscales. As test zone borders, laser-processed adhesive tape for biomedical applications purchased from 3M were used. Typically, an adhesive tape with laser-cut test zones was glued onto the corresponding substrate (PET, PET@toner or PS) and the as-obtained microwells were used for antibody immobilization. O_3_/UV treatment was carried out using ozone cleaner (Osilla, Sheffield, UK). Selective toner removal from test zones was accomplished by brief sonication (no more than 15 s) in an acetone, followed by subsequent rinsing with acetone and water and drying with compressed air.

#### 3.2.2. Passive Immobilization of Antibodies

The typical procedure of antibody immobilization consisted of dispensing 25 µL of an antibody solution onto the pre-prepared well. Capture antibody concentration of 5 µg/mL was typically used (pAb a-CRP as test antibodies and gIgG as negative control, respectively). Typically, 50 mM Na/carbonate buffer pH 9.6 or 50 mM Na/acetate buffer pH 5.0 (only for studies on the effect of pH on the effectiveness of antibody adsorption) was used as antibody coating medium. Substrates with dispensed coating solutions were then incubated at ambient temperature (~23 °C) for 2 h in a humid atmosphere. Test zones were then blocked against non-specific interactions by applying 25 µL of a 1% BSA solution in PBS + 0.05% Tween20 (PBST) for 30 min. To wash the test zones after blocking as well as during the immunoassay, PBST buffer was used.

### 3.3. Characterization

#### 3.3.1. Antibody Immunolabeling and Indirect Sandwich Assay

For the comparative assessment of the surface density of adsorbed antibodies, the immunolabeling procedure (direct ELISA) was employed. For this purpose, 25 µL of a-rIgG-ALP conjugate solution, 0.5 µg/mL, was dispensed into each test zone and incubated in a humid atmosphere for 30 min, after which microwells were washed thoroughly (at least three times) with PBST and dried in a stream of compressed air. Indirect sandwich ELISA was carried out in a similar way: first, 25 µL of mixture containing model antigen (hCRP, 100 ng/mL) and capture antibody (mAb a-CRP) in PBST + 1% BSA was incubated in each microwell for 30 min, followed by washing and dispensing 25 µL solution of a-mIgG-ALP conjugate, 0.5 µg/mL. Substrates were incubated in a humid atmosphere for 30 min and then thoroughly rinsed. All the antibody and conjugate solutions used in the immunoassay construction were in PBST with 1% BSA.

An amount of 10 mM *p*-nitrophenyl phosphate in 50 mM Na/carbonate buffer pH 9.6 was used for colorimetric detection. On each previously washed and dried test zone, 25 µL of substrate solution was simultaneously dispensed (using an automatic multichannel pipette) and incubated in a humid atmosphere until colors of the test zones and control zones were clearly differentiated by a naked eye (typically about 10 min). The solution of enzymatic reaction product was transferred (in a volume of 20 µL) from the test zone to the wells of a classic 96-well plate, and 80 µL of CB was added into each measured well. Spectrophotometric microtiter plate reader Magellan (Tecan) was used to measure the absorbance of *p*-nitrophenol (endpoint format, λ = 405 nm).

#### 3.3.2. Optical and Microscopic Analysis

Laser microscope LEXT OLS4000 (Olympus) was used to investigate the topography of PET film and toner, as well as to determine PET@toner surface profiles. Absorption spectra of dry foils in range 320–900 nm were recorded using a Multiskan Go spectrophotometer working in a microplate reader format (Thermo Scientific).

#### 3.3.3. Contact Angle Determination

Measurements of sessile drop contact angle were carried out using the static method and based on a drop shape analysis. Optical system consisted of a digital MIC-D microscope (Olympus) with an illuminator. The observed drops had a volume of 2 µL. Deionized water was used as a medium. The dedicated LB-ASDA software as part of ImageJ was used for results processing.

#### 3.3.4. Surface ζ-Potential Measurements

Surface ζ-potential (SZP) measurements were carried out according to the modified protocol of laser Doppler electrophoresis, using Zetasizer Nano ZS device equipped with a dedicated dip cell for surface ζ-potential. As anionic SZP tracer, colloidal solution of coffee creamer Coffee Mate (Nestle), 10 µg/mL in 50 mM Na/acetate buffer pH 5.0 and 50 mM Na/carbonate buffer pH 9.6 was used, according to the previously described protocol with slight modification [55]. PET, PET@toner, and PS samples with dimensions of 4 × 5 mm were glued to the table between the electrodes by means of a waterproof, double-sided adhesive tape. A series of ζ-potential measurements of the tracer at various distances from the surface (h: 125–875 µm, distance regulated using micrometer screw) allowed for a linear extrapolation of the ζ-potential value at the surface (h = 0µm) and the determination of ζ-potential of the surface according to Equation (1):


Surface Zeta Potential (SZP) = − intercept (h = 0 µm) + tracer ζ-potential
(1)

SZPs of PET, toner, and PS substrates after adsorption of pAb a-CRP were measured in an analogous manner as for uncoated substrates (according to the procedure described above, using Na/carbonate buffer pH 9.6 for antibodies adsorption and without additional surface blocking). All measurements were performed in triplicate.

## 4. Conclusions

Taking into account a number of interesting functionalities and advantages of the cured printer toner on flexible PET foil (excellent adhesion and covering properties, high hydrophobicity, facile removal), the possibilities of this substrate were thoroughly tested for the formation of immunoreceptor layers. It has been confirmed that irreversible, passive adsorption of antibodies is possible on PET@toner. It has been shown that increasing coverage of the PET foil with a layer of strongly hydrophobic laser toner both encourages total antibody adsorption efficiency and enhances the kinetics of the process. Immobilization on toner also improves the sensitivity of the sandwich assay; hence, the preservation of antibodies biological activity after binding was confirmed. Brief oxidation of the PET and toner surface with ozone/UV treatment and the associated hydrophilization give the surface permanent, non-fouling character, manifested by a decrease in the ability to bind antibodies over 70% and lasting for at least 7 days. In addition, laser printing at a properly selected grayscale level with the possibility of later removing the toner layer from the PET backing allowed for fine-tuning of both wettability and antibody binding capacity. The results benchmarked against pristine polystyrene indicate a better (by about 50%) antibody binding capacity of pristine PET and PET@toner substrates. Studies on the adsorption mechanism by surface zeta potential measurements and the influence of pH on antibody assembly processes indicate a key contribution of entropy-driven hydrophobic interactions for all types of tested substrates. We believe that the developed, versatile interphases based on PET@toner, depending on their configuration, can find applications as components for the cheap and easily available Lab-on-a-Foil microfluidic systems and sensing platforms.

## Figures and Tables

**Table 1:** Surface ζ-potential (SZP) values and relative surface densities of adsorbed pAb a-CRP (determined by immunolabelling) recorded for different substrates (PET, toner, PS) before and after antibody immobilization using coating buffers of different pH (50 mM carbonate buffer pH 9.6 and 50 mM acetate buffer pH 5.0, respectively). (*n* = 3). PET—poly(ethylene terephtalate), PS—polystyrene, Ab—antibody.

Substrate	Antibody Coating	SZP (mV)		Ab Surface Density (Normalized) (%)	
		pH 9.6	pH 5.0	pH 9.6	pH 5.0
**PET**	-	−48.5 ± 4.9	−17.7 ± 1.6	-	-
	+	−63.7 ± 6.9	−25.6 ± 1.5	100.0 ± 4.7	99.2 ± 4.1
**PET@toner**	-	−41.8 ± 3.1	−22.5 ± 1.7	-	-
	+	−58.8 ± 2.4	−27.0 ± 3.2	97.3 ± 3.9	82.8 ± 4.8
**PS**	-	−43.8 ± 3.2	−29.5 ± 1.7	-	-
	+	−28.6 ± 2.2	−33.6 ± 1.9	67.4 ± 3.5	66.4 ± 7.9

## Data Availability

Data available on request.

## Abbreviations

ALP	alkaline phosphatase
a-mIgG	*anti*-mouse IgG antibody
a-rIgG	*anti*-rabbit IgG antibody
BCIP/NBT	5-bromo-4-chloro-3-indolyl-phosphate/nitro blue tetrazolium
BSA	bovine serum albumin
CRP	C-reactive protein
ELISA	enzyme-linked immunosorbent assay
gIgG	immunoglobulin G from goat
mAb a-CRP	*anti*-CRP, monoclonal antibody
mIgG	immunoglobulin G from mouse
n-Bu	*n*-butyl
pAb a-CRP	*anti*-CRP, polyclonal antibody
PDMS	polydimethylsiloxane
PET@toner	poly(ethylene terephtalate) sheet covered with cured laser toner
PNNP	*p*-nitrophenyl phosphate
PS	polystyrene
PTFE	poly(tetrafluoroethylene)
SZP	surface ζ-potential
